# Interventional Occlusion of Large Patent Ductus Arteriosus in Adults with Severe Pulmonary Hypertension

**DOI:** 10.3390/jcm12010354

**Published:** 2023-01-02

**Authors:** Zeming Zhou, Yuanrui Gu, Hong Zheng, Chaowu Yan, Qiong Liu, Shiguo Li, Huijun Song, Zhongying Xu, Jinglin Jin, Haibo Hu, Jianhua Lv

**Affiliations:** 1 Department of Structural Heart Disease, Fuwai Hospital, State Key Laboratory of Cardiovascular Disease, National Center for Cardiovascular Diseases, Chinese Academy of Medical Sciences, Peking Union Medical College, Beijing 100037, China; 2 Department of Cardiology, Fuwai Hospital, State Key Laboratory of Cardiovascular Disease, National Center for Cardiovascular Diseases, Chinese Academy of Medical Sciences, Peking Union Medical College, Beijing 100037, China; 3 Department of Vascular Surgery, Fuwai Hospital, State Key Laboratory of Cardiovascular Disease, National Center for Cardiovascular Disease, Chinese Academy of Medical Sciences, Peking Union Medical College, Beijing 100037, China

**Keywords:** patent ductus arteriosus, pulmonary hypertension, transcatheter closure, risk factor, prognosis

## Abstract

(1) Background: the indications for transcatheter closure of large patent ductus arteriosus (PDA) with severe pulmonary hypertension (PH) are still unclear, and scholars have not fully elucidated the factors that affect PH prognosis. (2) Methods: we retrospectively enrolled 134 consecutive patients with a PDA diameter ≥10 mm or a ratio of PDA and aortic >0.5. We collected clinical data to explore the factors affecting follow-up PH. (3) Results: 134 patients (mean age 35.04 ± 10.23 years; 98 women) successfully underwent a transcatheter closure, and all patients had a mean pulmonary artery pressure (mPAP) >50 mmHg. Five procedures were deemed to have failed because their mPAP did not decrease, and the patients experienced uncomfortable symptoms after the trial occlusion. The average occluder (pulmonary end) size was almost twice the PDA diameter (22.33 ± 4.81 mm vs. 11.69 ± 2.18 mm). Left ventricular end-diastolic dimension (LVEDD), mPAP, and left ventricular ejection fraction (LVEF) significantly reduced after the occlusion, and LVEF recovered during the follow-up period. In total, 42 of the 78 patients with total pulmonary resistance >4 Wood Units experienced clinical outcomes, and all of them had PH in the follow-up, while 10 of them had heart failure, and 4 were hospitalized again because of PH. The results of a logistic regression analysis revealed that the postoperative mPAP had an independent risk factor (odds ratio = 1.069, 95% confidence interval: 1.003 to 1.140, *p* = 0.040) with a receiver operating characteristic curve cut-off value of 35.5 mmHg (*p* < 0.001). (4) Conclusions: performing a transcatheter closure of large patent ductus arteriosus is feasible, and postoperative mPAP was a risk factor that affected the follow-up PH. Patients with a postoperative mPAP >35.5 mmHg should be considered for targeted medical therapy or should undergo right heart catheterization again after the occlusion.

## 1. Introduction

Patent ductus arteriosus (PDA) is one of the most common congenital heart diseases (CHD) as it accounts for 10–16% of CHDs [1,2]. Since 1967, when Porstmann conducted the first transcatheter closure to treat PDA [3], scientists have enhanced the device and delivery systems that physicians use when performing transcatheter closures tremendously. Currently, factors that determine whether a physician should perform a transcatheter closure to treat an individual with a normal-size PDA are clear and definite, and transcatheter closure is the first treatment choice of cardiologists. However, challenges still exist when closing large PDAs, especially those >10 mm. Various difficulties, including insufficient device sizes and pulmonary hypertension (PH), considerably decrease the success rate of the procedure. The invention of an oversized device larger than the 18/16 mm mushroom PDA occluder made it possible for physicians to close large PDAs. However, a large PDA is always accompanied by severe PH, and the mean pulmonary artery pressure (mPAP) is usually >50 mmHg. Thus, the irreversible pulmonary vascular change caused by severe PH results in persistent PH during the follow-up period which elevates the poor prognosis risk.

Right heart catheterization (RHC) is the method most commonly used to assess hemodynamics. Qp/Qs are less accurate at predicting the prognosis; nevertheless, scholars regard pulmonary vascular resistance (PVR) as a useful index [4,5,6,7,8,9,10,11]. However, the samples used in previous studies were small or the PDAs of the enrolled patients were not large enough. The indicators that predict follow-up PH after the occlusion of large PDAs with severe PH remain unclear. Thus, we retrospectively examined patients with large PDAs who underwent interventional treatment from 2010 to 2020, analyzed the feasibility of interventional treatments for large PDAs, and found a clear predictor regarding the postoperative persistence of PH.

## 2. Materials and Methods

### 2.1. Patients

We recruited patients who were diagnosed with large PDAs and underwent interventional treatment from 2010 to 2020. All the patients underwent an X-ray, electrocardiography, and transthoracic echocardiography (TTE). Some patients underwent multislice computed tomography before surgery. Researchers at the Structural Heart Disease Department at Fuwai Hospital conducted this retrospective study. The procedures of this study followed the ethical guidelines of the Declaration of Helsinki, and the Ethics Committee of Fuwai Hospital approved this study. Additionally, we obtained informed consent from all the participants (2021-1452).

### 2.2. Inclusion and Exclusion Criteria

Currently, the classification of PDA according to diameter is unclear. We defined a PDA diameter ≥10 mm or a ratio of PDA and aortic >0.5 as a large PDA. We defined indications that a transcatheter closure should be performed as follows: (1) large PDA; (2) pulmonary hypertension; and (3) an audible cardiac murmur attributable to the PDA. The exclusion criteria were as follows: (1) endocarditis; (2) resistant pulmonary hypertension; and (3) accompanied by other heart diseases requiring surgical repair [12].

### 2.3. Right Heart Catheterization and Transcatheter Closure

All patients underwent fluoroscopy-guided procedures under local anesthesia. We punctured the right femoral artery and vein and inserted puncture sheaths. We performed routine right heart catheterization, and we inserted a 5 or 6 Fr MPA2 catheter into the pulmonary artery and right ventricle to measure the pulmonary arterial pressure. We calculated total pulmonary resistance (TPR) based on blood oxygen saturation. If the TPR was >4 Wood Units, the patient inhaled oxygen for 10 min, and we repeated the procedure to calculate the TPR. Then, we inserted a 5 Fr pigtail catheter into the aorta (AO) and performed an aortography. We observed and measured the shape and diameter of the ducts using an aortography. The device deployment procedure was as follows: we passed a 5 or 6 Fr MPA2 catheter across the PDA into the aorta, exchanged the extra-stiff wire, and passed the delivery sheath. We passed the delivery sheath into the aorta via the PDA from the femoral vein. We deployed the device using fluoroscopy and angiographic guidance. The size of the pulmonary end of the PDA occluder used was larger than 16 mm and was produced by Lifetech Scientific Co., Ltd. (Shenzhen, China) or Starway Medical Tec Co., Ltd. (Beijing, China). We repeated the aortography and measured the continuous pressure. Finally, we released the device under X-ray and TTE guidance after confirming the correct position. We measured the pressure when the patient was stable after we released the device.

### 2.4. Postoperative and Follow-Up Outcomes

All patients underwent TTE, X-ray, and ECG again at 1, 3, 6, and 12 months. We detected the position and residual shunt of the occluder using TTE. We performed an X-ray to measure the cardiothoracic ratio after the procedure. We evaluated the PAP and predicted the PH in all the patients using TTE. The PH diagnostic criteria that we used to evaluate the TTE were based on the 2022 ESC guidelines [13]. We recommended that the patients repeat the RHC at 6 months after closure if doing so was deemed possible based on their TTE results. We defined the follow-up outcome according to the PH assessed with TTE or RHC after the occlusion. We conducted a telephone follow-up with all the patients before we analyzed the data. The follow-up time was the length from the discharge to the time of the last follow-up result that we could collect. We defined clinical outcomes as the follow-up PH, heart failure (NYHA III or IV), and/or hospitalization caused by PH.

### 2.5. Statistical Analysis

We used SPSS version 26.0 (IBM), R version 4.2.0 software, and GraphPad Prism version 8.0 (GraphPad Software Inc., La Jolla, CA, USA) to calculate and illustrate the data. The continuous variables in this study are expressed as the mean ± standard deviation (SD) or as the median (IQR), and the categorical variables are expressed as numbers and percentages. We compared the continuous variables using the independent sample *t*-test or the Wilcoxon rank-sum test. We performed a risk factor estimation of the outcomes using a combination of the least absolute shrinkage selection operator (LASSO) and univariate or multivariate logistic regression to determine the association measures. We used the receiver operating characteristic curve to define the cut-off value. We set statistical significance at *p* < 0.05.

## 3. Results

### 3.1. Patient Characteristics

A total of 152 patients underwent transcatheter therapy. We excluded 11 patients with other congenital heart diseases that required surgical repair or staged operation and 2 patients with severe residual shunts. The procedure failed in 5 patients whose PAP did not decrease and who also experienced uncomfortable symptoms after the trial occlusion; because of this, we pulled the device back. Thus, we recruited 134 patients for this study. A total of 88 patients underwent RHC, and 78 had a TPR >4 Wood Units.

Table 1 presents the baseline characteristics of the patients. The results of the preoperative TTE indicated a large PDA with an accompanying left-sided heart enlargement in all the patients. The results of the X-ray suggested heart enlargement with an increased cardiothoracic ratio. Five patients had a trivial pericardiac effusion before the operation which may have been caused by heart failure and disappeared after closure. Nine patients had other congenital heart diseases, including three with patent foramen ovale, four with a small atrial septal defect (7, 5, 5, and 4 mm), and two with small ventricular septal defects (3 and 2 mm). Twelve patients participated in targeted medical therapy before and after closure.

### 3.2. Transcatheter Closure and Postoperative PAP

We measured PAP and aortic pressure (AP) at baseline in all the patients using a catheter. The average size of the occluder (pulmonary end) was 22.33 ± 4.81 mm and was almost twice the size of the PDA diameter (β = 1.88; 95% confidence interval [CI]: 1.682–2.081; R^2^ = 0.73; *p* < 0.001). The left ventricular end-diastolic dimension (LVEDD) showed significant shrinkage accompanied with a decrease in the left ventricular ejection fraction (LVEF) below the normal level (Table 2). After closure, the mPAP of all 134 patients for whom the procedure was successful decreased (40.99 ± 12.34 mmHg), and the mean AP increased (14.52 ± 10.40 mmHg) (Table 2). Although the LVEF decreased after the occlusion, the clinical symptoms were not significant without pericardial effusion or heart failure. All the patients were able to walk out of the hospital one or two days after the occlusion.

The preoperative mPAP (76.0 [67.0,83.0] mmHg vs. 86.0 [70.5,94.0] mmHg; *p* > 0.05) in women aged <45 years was similar to that of men, but the postoperative mPAP (32.0 [24.0,41.0] mmHg vs. 41.0 [32.0, 52.5] mmHg; *p* = 0.015) was lower in women aged <45 than in men. The average difference between the pre- and postoperative mPAP in women and men was 5.53 mmHg and 8.59 mmHg, respectively, and they were not significant (*p* > 0.05). Both the preoperative (22.68 ± 4.84 mm vs. 27.24 ± 5.30 mm, *p* < 0.001) and postoperative (22.38 ± 4.26 mm vs. 27.21 ± 5.38 mm; *p* < 0.001) RVEDD were smaller in women.

### 3.3. Follow-Up Data

**Figure 1:**
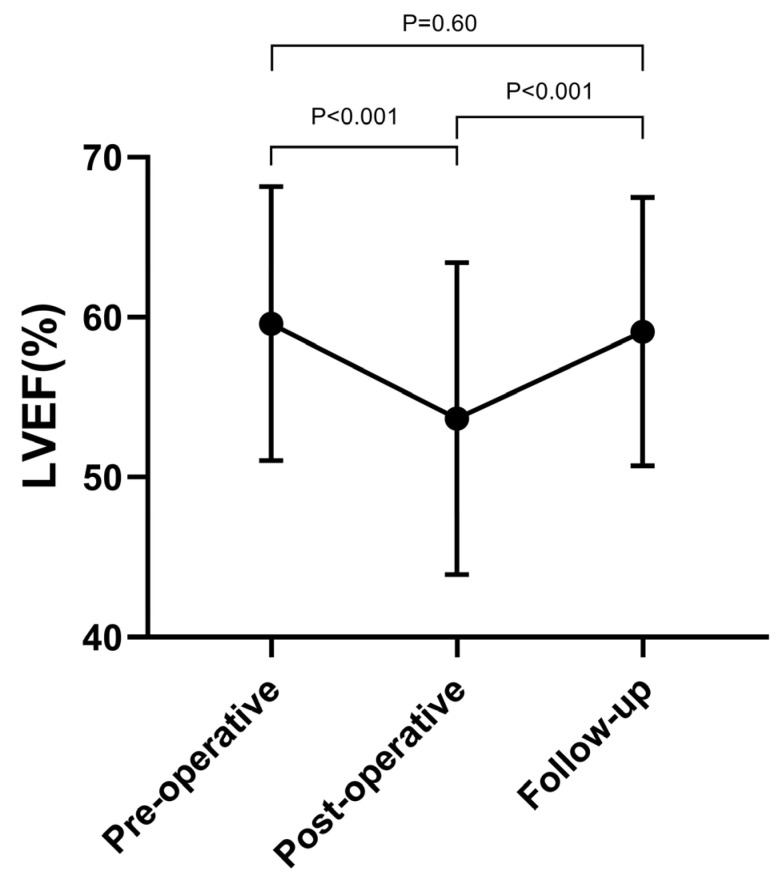
The postoperative LVEF was significantly reduced after occlusion and recovered to a normal level during the follow-up period. LVEF: left ventricular ejection fraction.

The average follow-up period for the 134 patients was 17.56 months. We conducted a follow-up TTE to assess the PAP, ventricle diameter, and LVEF. The follow-up LVEDD was normal and statistically different from the postoperative LVEDD (*p* < 0.001), and the RVEDD measured during the follow up was similar to the postoperative RVEDD (*p* > 0.05). However, the LVEF at the follow up was elevated compared with the postoperative LVEF (*p* < 0.001), and it gradually recovered to a normal level (*p* > 0.05) (Figure 1). Twelve patients who received targeted medical therapy stopped taking drugs and continued to have PH, but they were still alive at the final follow up.

### 3.4. Baseline of the Five Failed Patients

Although we did not include data from five failed patients, we compared them to the 78 successful patients who had a TPR >4 Wood Units. These five failed patients had a higher postoperative mPAP (62.20 ± 25.08 vs. 37.38 ± 11.23), a lower mPAP decrease (18.60 ± 22.50 vs. 41.94 ± 12.41), a lower Qp/Qs (1.58 ± 0.28 vs. 2.13 ± 1.04), a smaller LV diameter (52.60 ± 8.05 vs. 61.76 ± 9.27), and a higher LVEF (70.70 ± 7.05 vs. 61.16 ± 8.05). We created a logistic regression model based on these 83 patients and found that the mPAP after the occlusion was the only risk factor for closure failure (odds ratio [OR] = 1.115; 95% CI: 1.020–1.217; *p* = 0.016). The cut-off value of the mPAP was 49.5 mmHg, and the area under the curve (AUC) was 88.5% (*p* = 0.004).

### 3.5. Prognostic Value of the Post-Operative PAP after Occlusion

We assessed the PAP using TTE of 78 successful closure patients with a TPR >4 Wood Units, and only 5 patients repeated the RHC 6 months later. Forty-two patients had experienced clinical outcomes during the 12.00 (IQR:3.00-29.25, only 4 patients <3 months) months follow-up, and all of them were detected during the follow-up PH. Ten of them experienced heart failure, and four were hospitalized again because of PH. During follow-up, 42 patients (Group 1) still had PH, and 36 patients’ (Group 2) PAP had returned to normal. Group 1 had a higher postoperative mPAP and larger RVEDD during all periods (Table 3). Group 1 had a larger TPR and systolic Pp/Ps ratio than Group 2 before the occlusion (Table 3).

**Figure 2:**
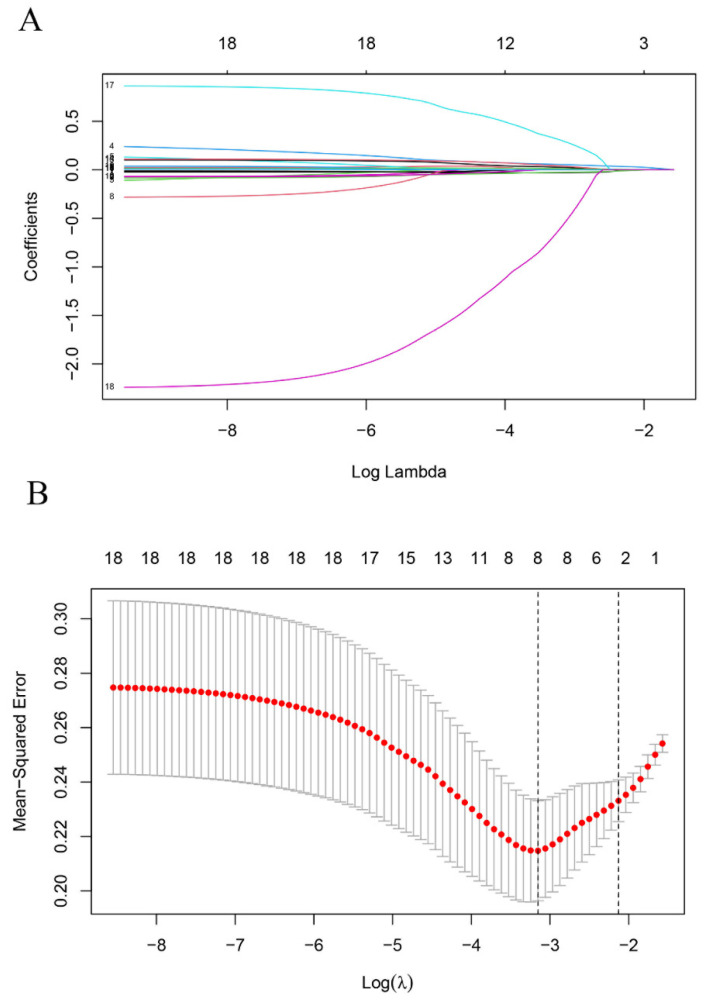
Least absolute shrinkage and selection operator (LASSO) analysis (**A**). We used LASSO regression to screen the prognostic factors. Coefficients of the determined characteristics are exhibited via lambda parameters (**B**). Cross validation indicated that three factors (postoperative mPAP, preoperative LVEDD, and postoperative systolic Pp/Ps) could be used in logistic regression. mPAP: mean pulmonary artery pressure; LVEDD: left ventricular end-diastolic dimension; Pp/Ps: pulmonary pressure, systemic pressure.

**Figure 3:**
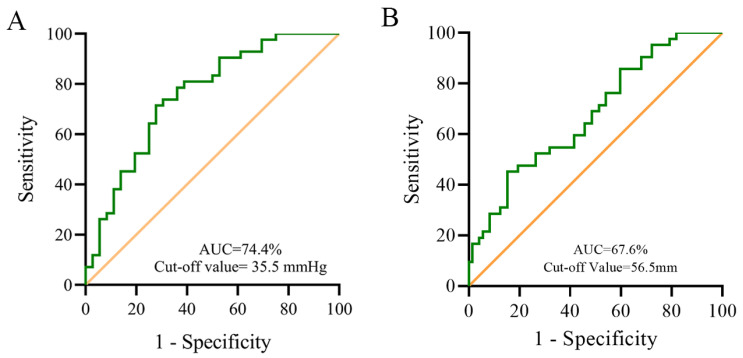
Receiver operating characteristic curve of postoperative mPAP (**A**) and LVEDD (**B**) for follow-up PH. AUC: area under the curve; mPAP: mean pulmonary artery pressure; LVEDD: left ventricular end-diastolic dimension.

Table 3 shows the potential risk factors for follow-up PH which we selected using the LASSO method (Figure 2). Table 4 shows the logistic regression analysis results. The postoperative mPAP was a risk factor that significantly affected the follow-up PH (*p* = 0.040, as shown in Table 4). We used the receiver operating characteristic (ROC) curve to determine the postoperative mPAP cut-off value to predict the PH, and we found that the cut-off value was 35.5 mmHg with an AUC of 74.4% (Figure 3A). The results of the logistic regression analysis showed that those with a higher PAP (>35.5 mmHg) had a higher risk of experiencing follow-up PH than those with a lower PAP (<35.5 mmHg) (OR = 5.682; 95% CI: 2.143–15.067; *p* < 0.001).

The LVEDD had no significant effect according to the multivariable regression model. However, when the regression model only included the preoperative factors, such as the baseline LVEDD, RVEDD, age, and sex, LVEDD was the only detected risk factor. Thus, we built an ROC curve, and the cut-off value was 56.5 mm with an AUC of 67.6% (Figure 3B). The results of the logistic regression analysis showed that those with a large LVEDD (>56.5 mm) had a lower risk of experiencing follow-up PH than those with a small LVEDD (<56.5 mm) (OR = 0.242; 95% CI: 0.083–0.703; *p* = 0.009).

## 4. Discussion

Presently, physicians still face difficulties when closing large PDAs, especially when treating patients with severe PH. Although there are different recommended closure strategies for different PVR grades, the ability to recognize the feasibility of closure and the postoperative persistence of PH is inconsistent across institutions. We analyzed and summarized the feasibility of large PDA interventional closures and found a clear predictor of PH postoperative persistence.

In small- or medium-sized PDAs, a device that is <16 mm can usually satisfy the clinical requirements. However, the PDA diameter in this study was ≥9 mm in all cases, and the size of the mushroom occluder was almost twice the PDA diameter. Thus, the size of the PDA occluder was larger than 16 mm. The normal-sized PDA could not provide sufficient support, and thus, the device was unstable and easily transposed under the large differential pressure of the AO and PA after implantation. Moreover, accurately measuring the diameters of the large PDAs was difficult, even with aortic angiography. After we implanted the device, the left disc of the device bulged into the aorta. Thus, the risk of obstructing the aorta still existed. Performing aortic angiography and measuring the continuous pressure from the aortic arch to the descending aorta below the device is necessary. The aorta and expanding pulmonary artery in adults can provide enough space for an oversized device; however, physicians still need to perform TTE and catheter examination with caution. We still do not recommend a transcatheter closure for a large PDA in children because of the higher risk of obstructing the descending aorta.

Many institutions are proficient with PDA transcatheter closure technology, but some questions still need special attention.

The first question concerns transcatheter closure criteria. The 2020 ESC Guidelines recommend that physicians should consider shunt closures for PDA patients with a PVR of 3–5 Wood Units and Qp/Qs >1.5 (IIa, C) and that physicians may consider a shunt closure after careful evaluation in a specialized center for patients with a PVR >5 Wood Units and Qp/Qs >1.5 (IIb, C) [14]. Compared with the 2015 ESC/ERS guidelines [15], this guideline attempts to make shunt closures more accessible for patients with over 5 Wood Units. This may benefit some patients with a higher PVR. Physicians in some clinical centers have adopted a trial occlusion strategy where some patients with >4 Wood Units undergo treatment. In this study, we adopted TPR, which is different from the recommended guidelines. Thus, when we set the inclusion criteria, we set the TPR dividing line to 4 Wood Units to control the PVR of patients at 3 Wood Units. In total, 78 patients with >4 Wood Units successfully implanted the PDA occluders, while their mPAP significantly decreased, and their heart function recovered. However, the closure eventually failed in five patients because of an increase in their pulmonary artery pressure and the accompanying uncomfortable symptoms after the occlusion. We could easily decide whether the occluder was suitable for release after observing clinical symptoms and analyzing the hemodynamics after the trial occlusion. One cannot deny that trial occlusions provide opportunities for patients with severe PH. 

The second question focuses on the LVEF change after the occlusion. In this study, we found that the LVEF in several patients was reduced to <30%, and no clinical symptoms were present according to the patients’ self-description. The LVEF reduced significantly after the closure, but it recovered after several months. LVEF is an index influenced by the preload and afterload. The preload decreases and the afterload increases after PDA treatment; therefore, the LVEF decreases after treatment. After several months, the cardiac reserve was gradually restored, and the LVEF and LV gradually recovered to a normal level. Therefore, the patient could return to daily life and perform physical labor several months after the operation. Ruth et al. reported the same result in children [16]. In practice, we observed that the change in the LVEF mostly happened in patients with a large PDA and LVEDD, and we predicted a significant reduction in the LVEF during the LV remodeling. A European study with a large sample revealed that the PDA diameter correlates well with postoperative LV dysfunction [17]. However, this was not significant in our study because we did not recruit patients with a normal PDA diameter.

The final question we should ask concerns PH prognosis after occlusion, especially in patients with TPR >4 Wood Units. For these patients, factors that affected follow-up PH were still unclear. Predicting follow-up PH could help physicians determine the subsequent treatment strategy, namely whether they should subsequently perform targeted medical treatment. Barst et al. suggested that Pp/Ps is appropriate to define PH, and Zhang et al. revealed that the postoperation systolic Pp/Ps ratio is a sensitive and specific parameter to identify postoperative PH [18,19]. However, the systolic Pp/Ps in our study was not significant in the multivariable regression model. The reasons may include the following. (1) The proportion of PH patients was different. Only 17 (12.6%) patients who participated in the previous study had PH during the follow up, whereas 42 (54%) patients who participated in our study had PH during the follow up. (2) Although the results of our study and the previous study showed that the systolic pulmonary pressure was similar in the baseline (115 ± 16 vs. 119 ± 18 mmHg), the postoperative systolic pulmonary pressure of the 42 follow-up PH patients who participated in this study was less than that of the participants who participated in the previous study (62 ± 15 vs. 85 ± 13 mmHg), and the mean pulmonary pressure decreased more significantly in the patients who participated our study. Thus, the average systolic Pp/Ps that we found was less than what the authors of the previous study found (44 ± 12% vs. 68 ± 15%). Therefore, postoperative systolic Pp/Ps was not significant in our study. However, the systolic Pp/Ps values between the patients with and without PH who participated in our study were also significantly different (43.95 ± 11.99% vs. 35.39 ± 9.50%; *p* < 0.001). This finding also indicates that the follow-up PH patients had a higher postoperative PAP.

We found that postoperative mPAP and mPAP differences were higher in the patients who had PH at follow-up than in those who did not have PH at follow-up. Postoperative mPAP was the only significant factor that affected the follow-up PH in patients with a TPR >4 Wood units. Using a logistic regression model, we found that those with a higher postoperative mPAP had a higher risk of having follow-up PH. The cut-off value suggested that a postoperative mPAP <35.5 mmHg was preferable. If the postoperative mPAP was greater than 35.5 mmHg, the patient was required to undergo targeted medical therapy or repeat the RHC 6 months later. As for the 12 patients who received targeted medical therapy, stopped taking the drugs, and were unwilling to undergo RHC again due to the economic burden, their prognosis may be relatively pessimistic. Many of these individuals were from developing countries and low-income populations. Cost efficiency is thus the largest concern when implementing RHC. Undergoing RHC is important regardless of whether patients with mild postoperative PH need to undergo targeted medical therapy. Thus, we are still recruiting a large sample population, and the pulmonary-pressure-based targeted medical therapy strategy is still under observation.

Although the LVEDD was not significant in the multivariable regression model, it was significant when we included only the preoperative factors in the model. This result was consistent with our clinical experience. The cut-off value of the LVEDD was similar to that found during clinical practice. Two reasons may explain this phenomenon: one is that a large RV compresses the LV and thus causes it to present a “D” shape on the TTE, and another is that the increased PAP changed the pre- and afterload of the left ventricle. A small LV may cause a patient to have a severe prognosis at follow-up. Physicians could even directly conduct targeted medical therapy without RHC in some circumstances. Therefore, patients with small LVEDD might need to have a further evaluation when undergoing this invasive operation. 

As for the 5 failed patients, we compared their clinical data to the data of the 78 successful patients, and the differences were statistically significant. In clinical practice, a postoperative mPAP >49.5 mmHg is always combined with occlusion failure, which was consistent with our findings. Although the sample was too small to have enough power to draw firm conclusions, the patients reported clinical symptoms of chest pain and dyspnea after the trial occlusion. This demonstrates that a trial occlusion is necessary and important when closing a large PDA.

This study has some limitations. First, it was a single-center retrospective study with inescapable referral bias. Second, we only recorded the terminal status of the patients over the telephone. Some early patients were missing local hospital TTE results or were absent from the examination for many years. Thus, collecting the exact time when the PAP returned to normal was difficult. Third, the patients who received targeted medicine therapy may have withdrawn from the therapy or only participated in half of it. Thus, evaluating the drug therapy efficiency was difficult. Fourth, over half of the patients did not declare whether they used diuretics and vasodilators in their medication records. Thus, we may have underestimated the PAP before closure. Fifth, 46 patients did not undergo the RHC. These patients’ PAP were similar to those of the patients with RHC, and the proportion of patients with >35.5 mmHg was not significantly different between these two populations (all *p* > 0.05). However, this finding was based on the experiences of our center and has certain limitations. Sixth, we measured the TPR in the past 10 years, which is different from what the guidelines recommend. Thus, when setting the inclusion criteria, we set the TPR dividing line as 4 Wood Units to control the PVR of patients at 3 Wood Units. Adopting the PVR would have been more appropriate. Finally, the TTE results obtained by the researchers at local clinical centers and our institution may have been inconsistent, and thus, the detected PH may have not been accurate. 

## 5. Conclusions

The closure of large PDA with severe PH is feasible with 96.4% success (5 out of 139 patients failed). However, mPAP over 35.5 mmHg after closure predicts follow-up PH and clinical outcomes that are likely worse. These patients should probably be treated with target therapy, and repeated RHC should be performed after PDA closure, optimally with PVR assessment. PVR (not TPR) should also be calculated before PDA closure. Patients with smaller LV before closure (less than 56.5 mm LVEDD) should be observed more carefully.

Unfortunately, the importance of PVR assessment before the closure and evaluation of an upper value of PVR that is safe for PDA closure cannot be derived from this study.

## Figures and Tables

**Table 1:** Baseline characteristics of patients.

Variables	N
Patients, n	134
Female, n (%)	98 (73.1)
Age, y	35.04 ± 10.23
BMI, kg/m^2^	20.43 ± 3.60
Heart function (NYHA)	
I, II	115
III, IV	19
Cardiothoracic ratio	0.60 ± 0.07
PDA diameter, mm	11.69 ± 2.18
Occluder size, mm	22.33 ± 4.81
Presence of other heart diseases, n	
PFO	3
Small ASD	4
Small VSD	2
Trivial pericardiac effusion, n	5
Complications, n	
Residual shunt	7
Femoral arteriovenous fistula	1
Systolic Pp/Ps ratio, %	82.93 ± 18.47
Targeted medical therapy, n	12
Endothelin receptor antagonists, n	7
Phosphodiesterase 5 inhibitors and guanylate cyclase stimulators, n	8
Prostacyclin analogues and prostacyclin receptor agonists, n	1

NYHA: New York Heart Association; ASD: atrial septal defect; BMI: body mass index; PDA: patent ductus arteriosus; PFO: patent foramen ovale; Pp/Ps: pulmonary pressure, systemic pressure.

**Table 2:** Pre- and postoperative variable comparison.

Variables	Preoperative	Postoperative	*p*
mPAP	76.79 ± 14.96	35.31 ± 12.05	<0.001
mAP	89.17 ± 12.23	103.70 ± 13.39	<0.001
RVEDD	24.22 ± 5.37	23.83 ± 4.86	>0.05
LVEDD	63.75 ± 10.05	58.53 ± 9.35	<0.001
LVEF	59.60 ± 8.11	52.94 ± 9.90	<0.001

mPAP: mean pulmonary artery pressure; mAP: mean arterial pressure; LVEDD: left ventricular end-diastolic dimension; LVEF: left ventricular ejection fraction; RVEDD: right ventricular end-diastolic dimension.

**Table 3:** Comparison of variables between patients with and without follow-up PH.

Variables	With Follow-Up PH (*n* = 42)	Without Follow-Up PH (*n* = 36)	*p*
Female, n (%)	32 (76.2)	25 (69.2)	0.61
Age, y	35.48 ± 9.97	33.81 ± 10.80	
Heart function (NYHA), n			0.21
I, II	34	33	
III, IV	8	3	
PDA diameter, mm	12.17 ± 2.52	12.14 ± 1.85	0.95
Occluder size, mm	23.29 ± 4.90	23.00 ± 4.67	0.79
Before occlusion at baseline			
Baseline TPR, Wood Units	12.95 ± 5.87	10.47 ± 4.77	0.047
Systolic Pp/Ps ratio, %	88.03 ± 17.40	81.40 ± 19.74	0.11
Qp/Qs	2.00 ± 1.18	2.29 ± 0.84	0.22
mPAP, mmHg	81.29 ± 11.32	78.14 ± 13.24	0.26
LVEDD, mm	58.95 ± 8.00	65.03 ± 9.67	0.003
RVEDD, mm	25.57 ± 5.61	22.89 ± 5.13	0.03
LVEF, %	61.40 ± 7.55	60.88 ± 8.70	0.78
Femoral artery SaO_2_, %	93.91 ± 3.52	94.42 ± 2.38	0.045
After occlusion at devices released			
Systolic Pp/Ps ratio, %	43.95 ± 11.99	35.39 ± 9.50	<0.001
mPAP, mmHg	41.69 ± 10.45	32.36 ± 10.07	<0.001
LVEDD, mm	54.81 ± 7.59	65.03 ± 9.67	0.023
RVEDD, mm	25.12 ± 5.26	22.53 ± 4.16	0.020
LVEF, %	55.28 ± 8.98	53.09 ± 8.70	0.28
mPAP difference before and after occlusion, mmHg	41.69 ± 10.45	32.36 ± 10.07	0.072

PH: pulmonary hypertension; PDA: patent ductus arteriosus; TPR: total pulmonary resistance; Pp/Ps: pulmonary pressure, systemic pressure.

**Table 4:** Univariable and multivariable logistic regression of persistent PH at follow-up.

Variables	Univariable (OR, 95% CI)	*p*	Multivariable (OR, 95% CI)	*p*
Postoperative mPAP	1.097 (1.040–1.158)	0.001	1.069 (1.003–1.140)	0.040
Preoperative LVEDD	0.921 (0.869–0.977)	0.006	0.958 (0.897–1.022)	0.195
Postoperative systolic Pp/Ps	1.083 (1.028–1.141)	0.003	1.027 (0.961–1.098)	0.428

PH: pulmonary hypertension; mPAP: mean pulmonary artery pressure; LVEDD: left ventricular end-diastolic dimension.

## Data Availability

Not applicable.
